# Supplementary material for: Severe Hypercapnia during Anaesthesia under Mechanical Ventilation in Two Paediatric Patients
Source: Animals (Basel). 2023 Feb 14;13(4):663. doi: 10.3390/ani13040663 (PMC9951664; doi:10.3390/ani13040663)
Supplement: Supplementary file 1 [file animals-13-00663-s001.zip › animals-2130034-supplementary.pdf]

animals MDPI

Table S1. Recorded values of V_T measured by the ventilator, and V_T, PIP, PEEP and C_D measured by the spirometer in case No 1.

Time Point	V_T (mL) Ventilator Setting	$V_{T\ INS}$/$V_{T\ EXP}$ (mL) on Spirometer	PIP/PEEP (cmH_2O) on Spirometer	C_D (mL/cmH_2O) on Spirometer	$ETCO_2$ (mmHg)
T_1	20	No recording	No recording		37–40
T_2	50	22/14	13/0	1.2	37–40
T_3	50			1.3	
T_4	50			1.9	116
T_5	Off	38/34	14–20/0		58

T_1 = start of VCV, after atracurium was given; T_2 = after increase in V_T setting on the ventilator; T_3 = after first recruitment manoeuvre (stepwise increase of PEEP up to 10 cmH_2O); T_4 = after second recruitment manoeuvre (PIP of 20 cmH_2O for 20 s); T_5 = manual ventilation.

Table S2. Recorded values of V_T measured by the ventilator, and V_T, PIP, PEEP and C_D measured by the spirometer in case No 2.

Time Point	V_T (mL) Ventilator Setting	$V_{T\ INS}$/$V_{T\ EXP}$ (mL) on Spirometer	PIP/PEEP (cmH_2O) on Spirometer	C_D (mL/cmH_2O) on Spirometer	$ETCO_2$ (mmHg)
T_1	45	25/20	8/0	2.2	71
T_2	50	25/20	9/0	2	95
T_3					197
T_4			12–25/0		
T_5					65

T_1 = start of VCV, after atracurium was given; T_2 = 25 min after atracurium; T_3 = after recruitment manoeuvre (PIP of 20 cmH_2O for 20 s with a PEEP of 5 cm cmH_2O after); T_4 = manual ventilation; T_5 = end of anaesthesia.
